# GLIDE Wellcome Open Research Gateway

**DOI:** 10.12688/wellcomeopenres.17985.1

**Published:** 2022-06-24

**Authors:** Jeffrey Kahn, Joseph Ali, Patricia Kingori, Maria Merritt, Claire Murray, Michael Parker

**Affiliations:** 1 Berman Institute of Bioethics, Johns Hopkins University, Baltimore, MD, 21208, USA; 2 Wellcome Centre for Ethics and Humanities, Oxford University, Oxford, UK

**Keywords:** Global Health, Global Health Ethics, Infectious Disease, Bioethics

## Abstract

The Global Infectious Disease Ethics (GLIDE) Collaborative is launching a new Wellcome Open Research (WOR) Gateway, and we as the leaders of GLIDE hope to encourage submissions to this timely and necessary new platform for publishing open access peer-reviewed articles focusing on this area.

## Editorial

It doesn’t require much argument to make the case that attention to ethics, infectious disease and global health has never been more important. The GLIDE Gateway represents a timely and necessary new platform for publishing open access peer-reviewed articles focusing on this area. GLIDE aims to provide a flexible collaborative platform for identifying and analyzing ethical issues arising in infectious disease treatment, research, response, and preparedness, through the lens of global health ethics. It has a focus on being inclusive of diverse global perspectives, and will pay particular attention to including the voices of researchers at all career stages. The idea for GLIDE pre-dated the onset of the coronavirus disease 2019 (COVID-19) pandemic as bioethics colleagues from the University of Oxford and Johns Hopkins University realized the value that collaboration could add to analyses, research, and advanced training related to ethics, policy, and infectious disease in the global health context. Fortunately, the Wellcome Trust agreed. We began working initially on a pilot project in 2019 and now are in the midst of a multi-year launch of the GLIDE collaborative platform with colleagues at Oxford, Johns Hopkins, and an International Expert Network.

As we carried out a series of virtual meetings over the last year with members of GLIDE, we heard multiple versions of a similar concern: there are many barriers to both publishing in and accessing the academic journal literature due to author publication costs and licence fees. The growth of open access publication has been a major step towards addressing the problem of accessing articles that otherwise reside behind paywalls, but author publication costs make the cost of publication prohibitive for many scholars across the globe and in particular for colleagues in low- and middle-income countries (LMICs) and early career scholars. These barriers are exacerbating the inequities surfaced during the pandemic, highlighting the importance of addressing structural obstacles to knowledge sharing. The GLIDE Gateway aims to be a small step in promoting much needed wider systemic change.

The GLIDE Gateway will provide a home for open access and fee-free publishing focusing on infectious disease and global health ethics and will create a living resource for cutting edge analysis and discussion in this increasingly important but overlooked area. The Gateway will be an inclusive space for diverse voices from across the globe, and create a community of scholars working on these topics. To attract high-impact submissions for publication, the Gateway will provide rapid peer-review by a community of committed scholars led by an advisory team including Profs. Michael Parker from Oxford and Jeffrey Kahn from Johns Hopkins.

In addition to its content area focus and participation from the growing GLIDE community, the Gateway will join other related Collections on Wellcome Open Research (WOR) from Epidemic Ethics and the Global Forum on Bioethics in Research, creating a growing presence on WOR for global health ethics content and offering the opportunity for WOR to become a leading source of global health ethics scholarship. For those committed to the principles of open access publication and free sharing of knowledge, the GLIDE Gateway represents a new option for publishing. With the help of the community, the GLIDE Gateway and the WOR platform will be recognized as a first-tier journal option for those working in global health ethics and infectious disease. To help seed the Gateway, presenters at this year’s Oxford Global Health Bioethics International Conference (now organized by GLIDE) with the theme “From Crisis to Wellbeing: Recognizing the Power and Potential of Global Health Ethics” will be encouraged to submit papers based on their presentations for publication on the Gateway.

In sum, the WOR GLIDE Gateway will provide a new opportunity for high-impact and quality, open access and free to publish literature, on global health ethics and infectious disease. It will be accessible and available to scholars from across the globe, with reduced barriers for those from LMICs. This approach “walks the talk” of open access publication and makes real the commitment to address some of the inequities in knowledge sharing created by traditional publishing models, all of which are critically important as the world struggles to emerge from a global pandemic. We welcome colleagues in the bioethics community to join us in this effort, to focus more research and scholarship on the issues of global health ethics and infectious disease, and to consider submitting articles to WOR, making our work more widely available. We will be glad to help facilitate your effort to publish in the Gateway.

## Data availability

No data are associated with this article.

